# Pyrrolidine dithiocarbamate reduces the progression of total kidney volume and cyst enlargement in experimental polycystic kidney disease

**DOI:** 10.14814/phy2.12196

**Published:** 2014-12-11

**Authors:** Michelle H. T. Ta, Padmashree Rao, Mayuresh Korgaonkar, Sheryl F. Foster, Anthony Peduto, David C. H. Harris, Gopala K. Rangan

**Affiliations:** 1 Michael Stern Laboratory for Polycystic Kidney Disease, Centre for Transplant and Renal Research, Westmead Millennium Institute, University of Sydney, Sydney, New South Wales, Australia; 2 Brain Dynamics Centre, Westmead Millennium Institute, Westmead Hospital, University of Sydney, Sydney, New South Wales, Australia; 3 Department of Radiology, Westmead Hospital and The University of Sydney, Sydney, New South Wales, Australia

**Keywords:** Bortezomib, inflammation, nuclear factor‐*κ*B, polycystic kidney disease, pyrrolidine dithiocarbamate

## Abstract

Heterocyclic dithiocarbamates have anti‐inflammatory and anti‐proliferative effects in rodent models of chronic kidney disease. In this study, we tested the hypothesis that pyrrolidine dithiocarbamate (PDTC) reduces the progression of polycystic kidney disease (PKD). Male Lewis polycystic kidney (LPK) rats (an ortholog of *Nek8/NPHP9*) received intraperitoneal injections of either saline vehicle or PDTC (40 mg/kg once or twice daily) from postnatal weeks 4 until 11. By serial magnetic resonance imaging at weeks 5 and 10, the relative within‐rat increase in total kidney volume and cyst volume were 1.3‐fold (*P *=**0.01) and 1.4‐fold (*P* < 0.01) greater, respectively, in LPK + Vehicle compared to the LPK + PDTC(40 mg/kg twice daily) group. At week 11 in LPK rats, PDTC attenuated the increase in kidney weight to body weight ratio by 25% (*P* < 0.01) and proteinuria by 66% (*P* < 0.05 vs. LPK + Vehicle) but did not improve renal dysfunction. By quantitative whole‐slide image analysis, PDTC did not alter interstitial CD68+ cell accumulation, interstitial fibrosis, or renal cell proliferation in LPK rats at week 11. The phosphorylated form of the nuclear factor (NF)‐*κ*B subunit, p105, was increased in cystic epithelial cells of LPK rats, but was not altered by PDTC. Moreover, PDTC did not significantly alter nuclear expression of the p50 subunit or NF‐*κ*B (p65)‐DNA binding. Kidney enlargement in LPK rats was resistant to chronic treatment with a proteasome inhibitor, bortezomib. In conclusion, PDTC reduced renal cystic enlargement and proteinuria but lacked anti‐inflammatory effects in LPK rats.

## Abstract (precis)

Lewis polycystic kidney rats were treated with pyrrolidine dithiocarbamate (PDTC) from weeks 4 to 11. Quantitative analysis of serial magnetic resonance images indicated that over time, the change in total kidney volume was 1.3‐fold higher in PDTC‐treated than in vehicle‐treated rats. PDTC treatment also decreased kidney weight to body weight ratio, renal cystic volume, and proteinuria.

## Introduction

Polycystic kidney diseases (PKD) are a group of genetically inherited disorders involving the formation of multiple renal cysts (Harris and Torres 2009; Halvorson et al. 2010). Autosomal Dominant PKD (ADPKD) arises due to mutations in *Pkd1* and/or *Pkd2* (International Polycystic Kidney Disease Consortium 1995; Mochizuki et al. 1996) and is characterized by the onset of symptoms in adulthood (Harris and Torres 2009). In Autosomal Recessive PKD (ARPKD), the mutation of *Pkhd1* usually causes lethality during fetal life or in early childhood (Onuchic et al. 2002; Harris and Torres 2009). Renal failure is one of the leading causes of mortality in PKD, and as there are no specific therapies available, eventually dialysis or renal transplantation is required (Halvorson et al. 2010).

The key histological features of PKD are the proliferation and dedifferentiation of cystic epithelial cells (CECs) accompanied by interstitial inflammation and fibrosis (Halvorson et al. 2010; Goilav 2011; Grantham et al. 2011; Norman 2011). Cyst enlargement, due to dysregulated transepithelial fluid secretion and CEC proliferation, leads to nephron obstruction and a gradual reduction in glomerular filtration rate (GFR; Grantham et al. 2011). Interstitial inflammation and fibrosis are also important factors that mediate cyst growth and the progression to end‐stage renal failure (Norman 2011; Ta et al. 2013). Multiple signaling pathways, including vasopressin‐cAMP, mammalian target of rapamycin (mTOR), and nuclear factor (NF)‐*κ*B pathways, are abnormally upregulated in PKD (Harris and Torres 2009; Qin et al. 2012). Because of the numerous cellular and signal transduction pathways involved, it has been suggested that a multi‐target therapeutic approach is needed to effectively suppress the progression to renal failure in PKD (Leonhard et al. 2011). Recent preclinical studies have demonstrated that single compounds with pleiotropic effects, such as curcumin (Leonhard et al. 2011) and triptolide (Leuenroth et al. 2007, 2008, 2010; Chen et al. 2014), slow the progression of cystic renal disease in rodent models.

Pyrrolidine dithiocarbamate (PDTC) is a heterocyclic dithiocarbamate derivative (Cvek and Dvorak 2007) which has consistently been reported to be protective in rodent models of chronic renal injury (Rangan et al. 2001; Theuer et al. 2002; Fujihara et al. 2007; Tapia et al. 2008; Ebenezer et al. 2009; Elks et al. 2009; Zhai et al. 2012) renal cancer (Morais et al. 2010) and other nonrenal diseases (Buac et al. 2012). In these studies, PDTC reduced cellular proliferation (Morais et al. 2006), inflammatory cell infiltration (Tamada et al. 2003), and proteinuria (Tapia et al. 2008), and these effects were correlated with the suppression of NF‐*κ*B, metal chelation, and antioxidant activity (Cvek and Dvorak 2007). However, to our knowledge, the efficacy of PDTC in PKD has not been investigated. Therefore, in the present study, we tested the hypothesis that chronic administration of PDTC attenuates the progression of cyst growth and interstitial inflammation and fibrosis, and reduces the decline in renal dysfunction in PKD. In addition, we assessed whether the proteasome inhibitor, bortezomib (BTZ), has similar effects to PDTC (Lovborg et al. 2006). To test the hypothesis, we utilized the Lewis polycystic kidney (LPK) rat, a *Nek8/NPHP9* ortholog (McCooke et al. 2012). In this model, cystic renal disease is characterized by diffuse collecting duct dilatation (phenotypically resembling human ARPKD), which arises at week 3 and increases with age (Phillips et al. 2007). LPK kidneys also display histological features that are typical of human PKD, including interstitial inflammation (weeks 6–12) and fibrosis (from week 12 onwards), and increased cell proliferation (which peaks at week 3 and declines thereafter; Phillips et al. 2007).

## Materials and Methods

### Animals

Animals were obtained from the LPK and Lewis rat colonies at the Animal Care Facility, Westmead Hospital and allowed ad libitum access to standard rat chow (20% protein, 4.8% fat, 4.8% crude fiber, 0.8% calcium, 0.7% phosphorus, 0.36% sodium, Specialty Feeds, Glen Forrest, WA, Australia) and water. All studies were approved by the Western Sydney Local Health District Animal Ethics Committee (Protocol 5103).

### Experimental design and dosing

#### Experiment 1

**Figure 1.**
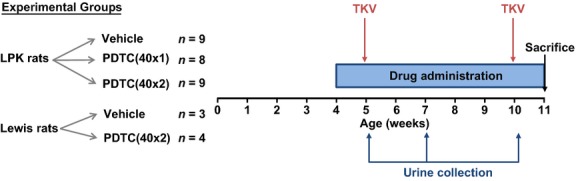
Outline of experimental design and study endpoints. Lewis polycystic kidney (LPK) and Lewis rats were administered intraperitoneal vehicle or PDTC once daily (40 × 1) or twice daily (40 × 2) beginning at postnatal week 4, and were euthanized at week 11. Urine was collected for measurement of renal function at weeks 5, 7, and 10, and MR imaging was performed at weeks 5 and 10. Abbreviations: TKV, total kidney volume measurement by MRI.

Four‐week old male LPK (*n* = 26) or Lewis rats (*n* = 7) were divided into five groups (Fig. 1): Group 1: LPK + vehicle (saline solution via intraperitoneal injection [i.p.i.]) (*n* = 9); Group 2: LPK + PDTC 40 mg/kg i.p.i. once daily (40 × 1) (*n* = 8); Group 3: LPK + PDTC 40 mg/kg i.p.i. twice daily (40 × 2) (*n* = 9); Group 4: Lewis + vehicle (*n* = 3); and Group 5: Lewis + PDTC (40 mg/kg twice daily, *n* = 4). Ammonium PDTC (Sigma–Aldrich, St. Louis, MO) was dissolved in sterile saline, filtered (0.45 *μ*m), and prepared daily. The dose and route were based on previous studies (Liu et al. 1999; Rangan et al. 1999). Side effects of PDTC included an immediate increase in motor activity, which peaked approximately 10 min postinjection, followed by mild sedation, hypersalivation, piloerection, and possible photosensitivity (suggested by a tendency to hide underneath the bedding). Similar effects have been observed in previous studies (Chabicovsky et al. 2010). Rats were euthanized at postnatal week 11 (i.e., after 7 weeks of study; Phillips et al. 2007) through deep anesthetization by an i.p.i. of ketamine: xylazine (20 mg/mL). Mid‐line laparotomy and nephrectomies were performed, kidneys were decapsulated, and excess blood was depleted from the heart.

#### Experiment 2

Four‐week old male LPK rats (*n* = 23) received either vehicle (saline) or BTZ (Velcade; Millennium Pharmaceuticals, Cambridge, MA, 0.2 mg/kg by i.p.i. twice weekly) from postnatal weeks 3 to 10. Animals were euthanized at week 6 (after 3 weeks of study, *n* = 3 per group) and week 10 (after 7 weeks of study, *n* = 8–9 per group). BTZ was prepared under sterile conditions and the doses were determined according to previous studies (Vogelbacher et al. 2010; Chen et al. 2012).

### Assessment of renal function and metabolic measurements

Urine was collected at weeks 5, 7, and 10, and serum was collected at the time of euthanasia (Rangan et al. 2013). Serum and urine were analyzed for albumin, protein, creatinine, and urea at the Institute of Clinical Pathology and Medical Research (Westmead Hospital) using VITROS slides (Ortho‐Clinical Diagnostics, Buckinghamshire, U.K.). Creatinine clearance was calculated by the formula, CrCl = (Urine Cr [*μ*mol/L] × Urine volume [mL/min])/Serum Cr (*μ*mol/L) corrected for body weight.

### Serial assessment and quantification of total kidney and cyst volume by MRI

Serial renal MRI scans were performed at weeks 5 and 10 on a subset of randomly selected LPK littermates [*n* = 4, vehicle group, and *n* = 4 from PDTC(40 × 2)] under isofluorane anesthesia, as previously described (Rangan et al. 2013). MRI data were acquired on a 3T GE Twinspeed Signa HDxT MRI system in combination with a transmit/receive coil (Mayo Clinic Medical Devices) at the Department of Radiology, Westmead Hospital. These T2‐W 3D FIESTA datasets were obtained with the following parameters: Coronal: FOV = 10, TE/TR = 4.1/12.1 msec, Flip = 45°, 352*256 acq. matrix, R/L Freq direction, 32 contiguous slices with slice thickness 0.8 and 2 NEX, Axial**:** FOV = 9 mm, TR/TE = 13.7/4.3 msec, all other parameters as for Coronal. Imaging data were then transferred to a PC in Digital Imaging and Communications in Medicine (DICOM) format and imported into 3D Slicer analysis software (http://www.slicer.org, Fedorov et al. 2012). On coronal images, regions of kidney tissue were labelled and subsequently assembled into a 3D model using the software Model Maker function, giving the output as an absolute kidney volume in mm^3^.

For analysis of cyst volume, the 2D kidney area segments (generated from the TKV analysis) were overlaid on their corresponding 2D MR images. A region‐based threshold method was used: from a histogram of signal intensities, an intensity threshold was chosen to segregate the cyst area from the parenchymal area within each 2D kidney segment. The intensity threshold was set on a case‐by‐case method, as previously described (Reichardt et al. 2009). The total kidney volume was calculated in Matlab (v7.10.0, 2010; The MathWorks, Inc., Natick, MA) by summing the product of kidney area labels and the slice thickness. The cyst volume was generated by applying the selected intensity threshold to all 2D segments within the kidney.

### Histology and quantitative whole‐slide image analysis

Coronal slices of kidney were fixed in either 10% formalin or methyl Carnoy's solution for 24 h, and sections (4 *μ*m in thickness) were deparaffinized. For morphological assessment, methyl Carnoy's‐fixed sections were stained with Periodic Acid Schiff (PAS). For immunohistochemistry, antigen retrieval was performed by microwave oven heating (for formalin slides only, 80% power, 25 min in 1x Antigen Decloaker; Biocare Medical, Concord, CA). Sections were blocked with 10% goat serum, and incubated with primary antibodies for 1 h at room temperature. Negative controls were incubated with 10% goat serum but not the primary antibody. The primary antibodies used were: (1) anti‐Ki67 (1:100, ab16667; Abcam, Cambridge, U.K.) to assess proliferation; (2) anti‐ED‐1 for CD68+ monocytes (1:400, MCA341R; Serotec, Kidlington, U.K.) to assess inflammation; (3) anti‐*α* smooth muscle actin (*α*SMA) to assess myofibroblasts accumulation (and also a marker of vascular smooth muscle cells) (1:4000, A2547; Sigma–Aldrich, St. Louis, MO); (4) anti‐phosphorylated‐p105 (P‐p105) to assess NF‐*κ*B expression (1:50, #4808; Cell Signaling Technology, Danvers, MA); and (5) anti‐p105/50 (1:100, P19838; Epitomics, Burlingame, CA) to assess NF‐*κ*B expression. Secondary biotinylated antibodies were applied for 30 min (anti‐mouse, 1:200, 65‐6440; anti‐rabbit, 1:200, 65‐6140; Life Technologies, Carlsbad, CA). Vectastain ABC reagent (Vector Laboratories, Burlingame, CA) was applied for 20 min, followed by diaminobenzidine. Sections were counterstained with methyl green (Sigma–Aldrich) and then dehydrated. To assess interstitial fibrosis, Sirius Red staining was performed on methyl Carnoy's fixed sections with 0.1% Direct Red 80 and 0.1% Fast Green FCF (Sigma–Aldrich) for 24 h.

To quantify immunohistology, whole slide digital images (20× magnification) were acquired using a scanner (Scanscope CS2, Aperio, CA). The percentage cyst area was analyzed on PAS slides using the positive pixel algorithm in Aperio ImageScope (v11.2.0.780, Leica Biosystems, Wetzlar, Germany). The following formula was applied: %cyst area = 100 × [(area of analyzed section − Total detected pixels)/area of analyzed section]. Immunohistochemistry for Ki67, CD68, Sirius Red, and *α*SMA was assessed using Leica SlidePath Digital Image Hub software (Leica Microsystems, Buffalo Grove, IL). By manually selecting pixels of interest, a color definition file was constructed for each stain, and the parameters of this file were then applied to the entire slide. For Ki67 and *α*SMA slides, the positive pixel algorithm was applied. For CD68 and Sirius Red slides, the measure stained area algorithm was used, and the percentage stained area was calculated by: 100 × positive area/total tissue area. The intensity threshold was adjusted to analyze slides which had a high background staining. The uroepithelium, renal pelvis, and cortical/medullary blood vessels were excluded from all immunohistochemistry analyses. For quantification of p50 staining, areas of cortex and the outer medulla were analyzed in Aperio ImageScope using the positive pixel algorithm, and data regarding the no. of positive (Np), weakly positive (Nwp), strongly positive (Nsp), and negative (Nn) pixels were obtained. Two different formulas were applied: (a) %p50 staining/tissue = 100 × (Nsp + Np)/(Nsp + Np + Nwp); and (b) %p50 staining/total area = 100 × (Nsp + Np)/(Nsp + Np + Nwp + Nn; i.e., including cystic area).

### Extraction of nuclear and cytoplasmic proteins, Western blotting and NF‐*κ*B binding assay

Extraction of nuclear protein from kidney homogenates (60–100 mg) was performed using two methods. First, a kit (NE‐PER^®^ reagents; Thermo Pierce, Rockford, IL) was used according to the manufacturer's instructions. Because the protein yield using this method was low, a second method (as described previously (Rangan et al. 1999)) was used and provided appropriate amounts of protein. Both the resulting cytoplasmic extract and nuclear extracts were then stored at −80°C. Protein concentration of the nuclear extracts was assessed using the DC Protein Assay (Bio‐Rad Laboratories, Hercules, CA).

For Western blotting, nuclear extracts were electrophoresed on 4–15% Mini‐PROTEAN TGX gels and semi‐dry transferred to PVDF membranes (Bio‐Rad). Membranes were blocked with Odyssey blocking buffer (LI‐COR Biosciences, Lincoln, NE), then incubated with antibodies for p105/50 (1:1000, P19838; Epitomics) and *β*‐actin (1:2000, #4970; Cell Signaling) overnight at 4°C, followed by secondary fluorescent antibodies (1:15000, #5366, #5257; Cell Signaling). Blots were imaged using the Odyssey infrared system and quantified using Odyssey software v3.0 (LI‐COR), and normalized using *β*‐actin.

NF‐*κ*B binding was assessed using a p65 transcription factor assay kit (10007889; Cayman Chemical, Ann Arbor, MI). Samples were added to a 96‐well plate coated with consensus double‐stranded DNA for NF‐*κ*B, incubated with NF‐*κ*B primary antibody overnight at 4°C, followed by horseradish peroxidase‐conjugated secondary antibody. The plate was developed and absorbance was measured at 450 nm. Positive controls (TNF‐*α*‐stimulated HeLa cell extract) were included, and binding specificity was assessed using competitor DNA.

### Statistical analysis

All statistical analyses were performed in SPSS for Windows, Version 16.0 (SPSS Inc., Chicago, IL). As the data were not normally distributed, nonparametric tests were applied. Differences between groups were analyzed using the Mann–Whitney *U* test (for two independent groups) or the Kruskal–Wallis test (for multiple groups), followed by the appropriate post hoc tests. *P*‐values < 0.05 were considered as statistically significant. Logarithmic transformation of the MRI volumes was performed to stabilize variances prior to statistical analysis. Repeated measures analysis of variance (ANOVA) was performed with time as the 2‐level within‐subject factor, and treatment as the between‐subject factor. The time × treatment interaction term tested whether the within‐rat change over time differed depending on the treatment group.

## Results

### Effects of PDTC on body weight and kidney enlargement

#### Animal health and body weight

Treatment with PDTC was well tolerated in Lewis and LPK rats and there was no mortality during the study. All treatment groups gained body weight at the same rate and there was no difference in body weight among groups at the end of the study (Table 1).

**Table 1.** Effects of PDTC on body weight, renal function, and proteinuria in Lewis and LPK rats.

Parameter	Lewis+V *N* = 3	Lewis+P(40 × 2)*N* = 4	LPK+V*N* = 9	LPK+P(40 × 1)*N* = 8	LPK+P(40 × 2)*N* = 9
BW at week 11 (g)	234 ± 6	233 ± 17	223 ± 20	218 ± 9	216 ± 20
CrCl/BW (fold‐change vs. Lewis+V)	1 ± 0.1	0.8 ± 0.2	0.5 ± 0.1^*^	0.5 ± 0.1	0.5 ± 0.2
Urine Pr:Cr (mg/mmol)					
Week 5	ND	ND	3 ± 6	19 ± 49	4 ± 7
Week 7	15 ± 26	ND	12 ± 13	8 ± 13	55 ± 95
Week 10	2 ± 4	20 ± 24	254 ± 166^**^	108 ± 52	87 ± 52^†^
24‐h urine volume (mL)					
Week 5	2 ± 1	5 ± 2	2 ± 1	3 ± 1	3 ± 1
Week 7	10 ± 4	10 ± 3	10 ± 4	9 ± 3	10 ± 5
Week 10	6 ± 2	8 ± 4	25 ± 4^**^	24 ± 8	22 ± 5
Serum urea (mmol/L)	10 ± 6	8 ± 2	15 ± 2	15 ± 4	15 ± 4
Serum Cr (*μ*mol/L)	25 ± 2	31 ± 8	46 ± 5^*^	49 ± 9	46 ± 9
Serum Albumin (g/L)	27 ± 4	27 ± 2	26 ± 2	23 ± 2^‡^	25 ± 2
HW:BW (%)	3.3 ± 0.3	3.0 ± 0.1	4.8 ± 0.3^**^	5.2 ± 0.4	4.6 ± 0.6

BW, body weight; Cr, creatinine; CrCl/BW, creatinine clearance corrected for body weight; HW:BW, heart weight to body weight ratio; LPK, Lewis polycystic kidney; ND, not detected; P(40 × 1), PDTC 40 mg/kg once daily; P(40 × 2), PDTC 40 mg/kg twice daily; Pr:Cr, urinary protein to creatinine ratio; V, vehicle.

Data expressed as mean ± SD.

*P < 0.05 versus Lewis + vehicle, **P < 0.01 versus Lewis + vehicle, ^†^*P* < 0.05 versus LPK + vehicle, ^‡^*P* < 0.01 versus LPK + vehicle.

#### Assessment of TKV and cyst volume by MRI

**Figure 2.**
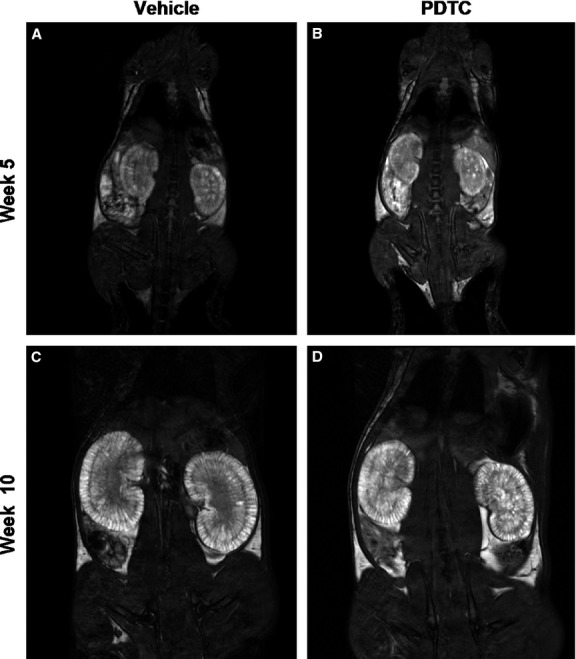
Representative T2 weighted MR images of kidneys from Lewis polycystic kidney (LPK) rats. Shown in the top panel are images captured at week 5, of vehicle‐treated (A) and PDTC‐treated (B) animals. The bottom panel shows images from week 10 of vehicle‐treated (C) and PDTC‐treated (D) animals.

**Figure 3.**
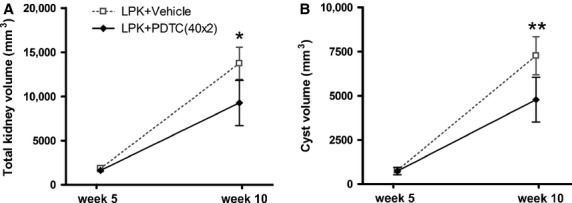
Effect of PDTC on mean TKV (A) and absolute cyst volume (B) in Lewis polycystic kidney (LPK) rats at weeks 5 and 10. Compared to PDTC treatment, vehicle treatment resulted in a significantly higher within‐rat change in TKV and absolute cyst volume. *P = 0.01 versus LPK + Vehicle, **P < 0.01 versus LPK + Vehicle.

At week 5, MRIs showed that the kidneys of LPK rats were reniform in shape and enlarged (Fig. 2A and B). On axial and coronal T2‐weighted MR images, the kidney cortices of LPK rats were hyperintense compared to normal kidneys, indicating a higher fluid content. At week 10, LPK kidneys retained the reniform shape but were increased in size compared to week 5 (Fig. 2C and D). At this time point, the cortex was hyperintense, along with multiple, loculated hyperintense regions throughout the inner and outer medulla, suggesting that cystic dilatations had developed in the collecting ducts. By quantitative analysis, the relative within‐rat increase in TKV from week 5 to week 10 was 1.3‐fold greater in the LPK + vehicle group compared to LPK + PDTC (40 × 2) (95% CI 1.10–1.60‐fold, *P* = 0.010, Fig. 3A). In addition, the relative within‐rat increase in absolute cyst volume from week 5 to week 10 was 1.4‐fold higher in the LPK + vehicle group compared to LPK + PDTC(40 × 2) (95% CI 1.20–1.72‐fold, *P* < 0.01, Fig. 3B).

#### Kidney to body weight ratio (KW:BW)

**Figure 4.**
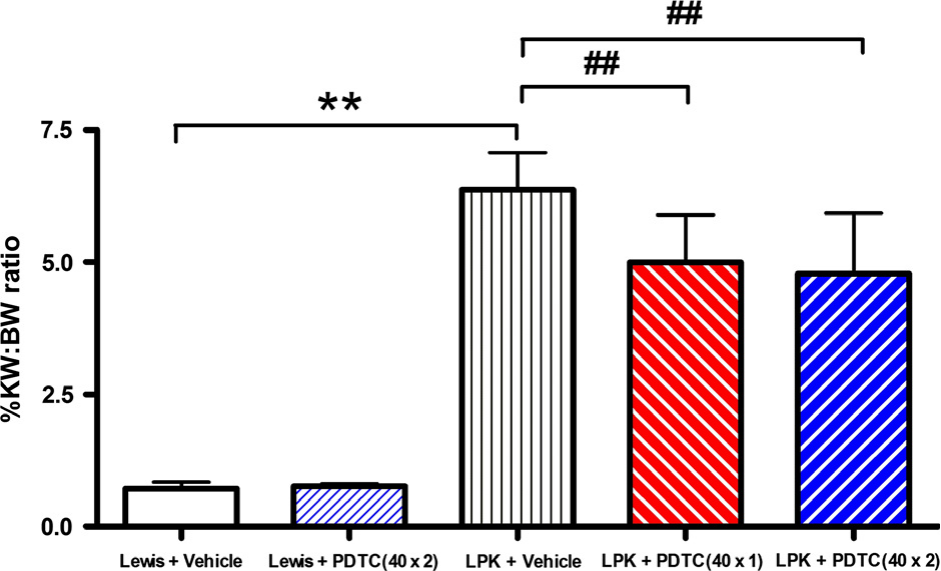
Effect of PDTC on KW:BW at week 11. The KW:BW was 9‐fold higher in Lewis polycystic kidney (LPK) compared to the Lewis groups, and PDTC treatment decreased KW:BW by 25% in LPK rats. Data as Mean + SD, **P < 0.01 versus Lewis + Vehicle, ##*P* < 0.01 versus LPK + Vehicle.

At the time of euthanasia, LPK kidneys were enlarged, pale, and fluid‐filled. The KW:BW in vehicle‐treated LPK rats was increased 9‐fold compared to vehicle‐treated Lewis rats (LPK + vehicle 6.4 ± 0.7, vs. Lewis + vehicle 0.7 ± 0.1%, *P* < 0.01, Fig. 4). The KW:BW was reduced by 22% and 25% in the PDTC(40 × 1) and PDTC(40 × 2) groups, respectively, compared with vehicle‐treated LPK rats (*P* < 0.01 vs. LPK + Vehicle).

### Effects of PDTC on proteinuria and renal function

#### Proteinuria

At weeks 5 and 7, the urine protein to creatinine ratio (Pr:Cr) was similar in all groups (Table 1). At week 10, Pr:Cr was increased in vehicle‐treated LPK rats compared to the Lewis group (*P* < 0.01). Treatment with PDTC in LPK rats attenuated proteinuria by 66% in the LPK + PDTC(40 × 2) group compared to LPK + vehicle (*P* < 0.05). A trend for a reduction in proteinuria was also observed in the LPK + PDTC(40 × 1) group (*P* = 0.10 vs. LPK + vehicle), which was still elevated compared to Lewis + vehicle.

#### Renal function

LPK rats had higher final mean serum creatinine compared to Lewis rats (*P* < 0.05, Table 1). Creatinine clearance (adjusted for body weight) was approximately 50% lower in LPK + vehicle than Lewis + vehicle at week 10, but this was not altered by PDTC treatment. No differences in serum urea were found amongst the groups (Table 1). At week 10, LPK rats had an approximately 4‐fold greater mean 24‐h urine volume than Lewis animals (*P* < 0.01, Table 1), but this was not attenuated by PDTC.

### Effects of PDTC on renal histology

#### Kidney histology

**Figure 5.**
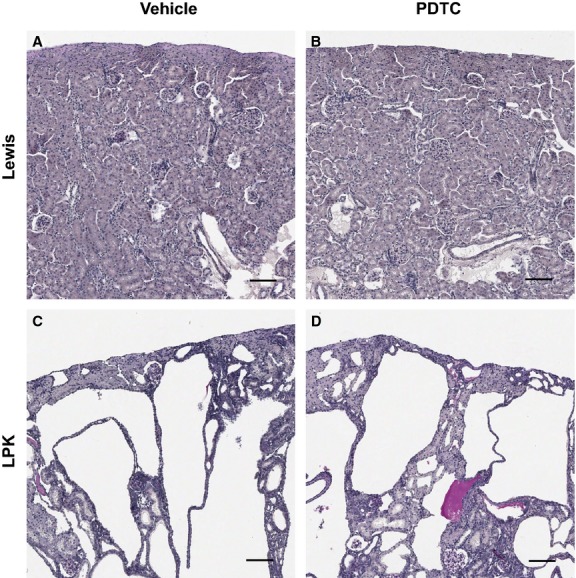
Periodic Acid Schiff (PAS) stained kidney cortices from Lewis rats treated with vehicle (A) and PDTC(40 × 2) (B), and Lewis polycystic kidney (LPK) rats treated with vehicle (C) and PDTC(40 × 2) (D). Compared to Lewis kidneys, LPK kidneys displayed diffuse cystic disease at week 11, characterized by collecting duct dilatation and interstitial injury. No significant differences in percentage cyst area were observed in the PDTC‐treated groups compared to vehicle‐treated LPK. Scale bar = 100 *μ*m.

In LPK animals, cystic lesions were diffuse throughout the cortex and outer medulla, and commonly displayed an ellipsoid or oblong morphology (Fig. 5C). Smaller tubular dilatations were observed in the inner medulla. Cortical glomeruli displayed expanded Bowman's spaces. Qualitative microscopic assessment of PAS‐stained sections did not reveal any differences between treated and untreated LPK animals, in morphology, or in the proportions of interstitial and cystic areas (Fig. 5C and D). By whole‐slide quantitative analysis, cystic areas accounted for approximately 50% of the total section area in LPK kidneys, and the percentage cyst area was not attenuated with PDTC treatment (Table 2).

**Table 2.** Effects of PDTC on renal histology in Lewis and LPK rats.

Histological parameter	Lewis+V *N* = 3	Lewis+P(40 × 2) *N* = 4	LPK+V *N* = 9	LPK+P(40 × 1) *N* = 8	LPK+P(40 × 2) *N* = 9
%Cyst area	–	–	52.2 ± 7.3	51.1 ± 8.2	56.0 ± 5.9
Cell proliferation (%Ki67)	1.0 ± 0.0	1.0 ± 0.0	4.6 ± 2.9^*^	4.1 ± 1.8	4.6 ± 2.1
Interstitial monocyte accumulation (%CD68)	0.5 ± 0.2	0.2 ± 0.1	5.3 ±1.9^**^	5.4 ± 2.0	6.1 ± 2.7
Interstitial collagen deposition (%Sirius Red)	3.8 ± 1.3	3.3 ± 2.0	16.0 ± 7.3^**^	13.6 ± 4.7	11.2 ± 5.0
Interstitial myofibroblasts (%*α*SMA)	1.7 ± 0.6	2.0 ± 0.0	7.8 ± 3.8^**^	6.6 ± 3.5	8.8 ± 5.0

LPK, Lewis polycystic kidney; P(40 × 1), PDTC 40 mg/kg once daily; P(40 × 2), PDTC 40 mg/kg twice daily; V, vehicle.

Data expressed as mean ± SD.

*P < 0.05 against Lewis + Vehicle, **P < 0.01 against Lewis + vehicle.

#### Cell proliferation

In Lewis animals, Ki67 stained occasionally in selected proximal tubule nuclei and glomerular cells. In contrast, Ki67 staining was more diffuse and intense in LPK rats, particularly in the nuclei of outer medullary CEC and interstitial cells. Ki67+ nuclei were also present in epithelia of inner medullary dilated tubules, cortical proximal tubules and some cortical CECs. Whole‐slide quantitative analysis showed that Ki67 positivity was significantly higher in vehicle‐treated LPK compared to Lewis rats (*P* < 0.05), but this was not affected by PDTC (Table 2).

#### Interstitial monocyte and myofibroblast accumulation

In Lewis kidneys, there were few CD68+ cells in the cortical and outer medullary interstitium. In contrast, LPK kidneys demonstrated diffuse CD68 positivity throughout the outer medullary interstitium, with sparser and less intense staining in the inner medulla and cortex. By whole‐slide analysis, CD68 positivity was greater in LPK compared to Lewis animals (*P* < 0.01, Table 2), but not affected by PDTC. In Lewis animals, *α*SMA was only apparent in vascular smooth muscle and selected medullary rays. In LPK animals, *α*SMA staining was diffuse, occurring predominantly in the outer medulla and in areas of neoangiogenesis. By quantitative analysis, the increase in *α*SMA positivity was not altered by PDTC (*P* < 0.01, Table 2).

#### Interstitial fibrosis

In Lewis animals, collagen deposition (measured by Sirius Red staining) occurred only in periarteriolar areas. In LPK animals, collagen was primarily deposited in the outer medullary interstitium and localized to peritubular and pericystic regions. Compared to Lewis, LPK rats had 4‐fold higher Sirius Red positivity (*P* < 0.01), but this was not altered by PDTC treatment (Table 2).

### Effects of PDTC on renal p50 expression and activation

#### Localization of P‐p105

**Figure 6.**
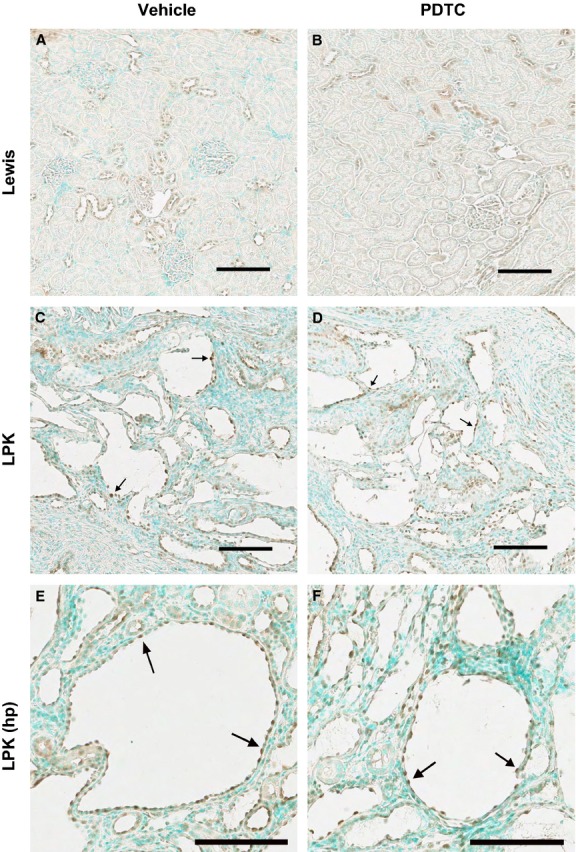
Effect of PDTC on cortical P‐p105 immunolocalization. Shown are kidney cortices from Lewis rats treated with vehicle (A) and PDTC(40 × 2) (B), and LPK rats treated with vehicle (C, E) and PDTC(40 × 2) (D, F). Lewis cortices displayed P‐p105 positivity in distal tubule nuclei. In LPK kidney cortices, P‐p105 stained strongly in cystic epithelial cell (CEC) cytoplasm and nuclei (arrows). Scale bar = 100 *μ*m.

To assess NF‐*κ*B activation, we examined the expression of the classical NF‐*κ*B protein p50/p105. The p105 protein is cleaved during NF‐*κ*B activation to produce p50 (Salmerón et al. 2001; Hayden and Ghosh 2012). In this process, p105 becomes phosphorylated, and thus the phosphorylated form (P‐p105) is a marker of NF‐*κ*B activation. In Lewis kidneys, P‐p105 staining was diffuse and intense in the nuclei of inner medullary collecting duct epithelia (Fig. 6A), whereas in the cortex it stained only weakly in selected distal tubules. In LPK kidneys, P‐p105 was predominantly located in the cytoplasm and nuclei of CECs. Positive CEC staining was detected in all regions of the kidney, but was most diffuse and intense in the cortex (Fig. 6C). P‐p105 was also weakly present in cortical distal tubules. Qualitative microscopic assessment found no changes in P‐p105 staining with PDTC (Fig. 6B and D).

#### Expression and localization of p50

**Figure 7.**
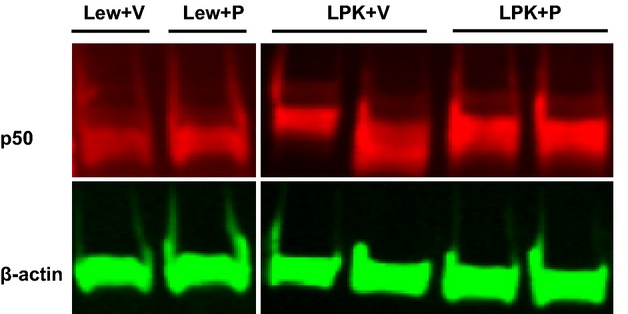
Effect of PDTC on p50 expression in nuclear extracts from whole‐kidney homogenates shown are representative Western blots for p50, with *β*‐actin as the loading control. Abbreviations: Lew, Lewis; P, PDTC(40 × 2); V, Vehicle.

**Figure 8.**
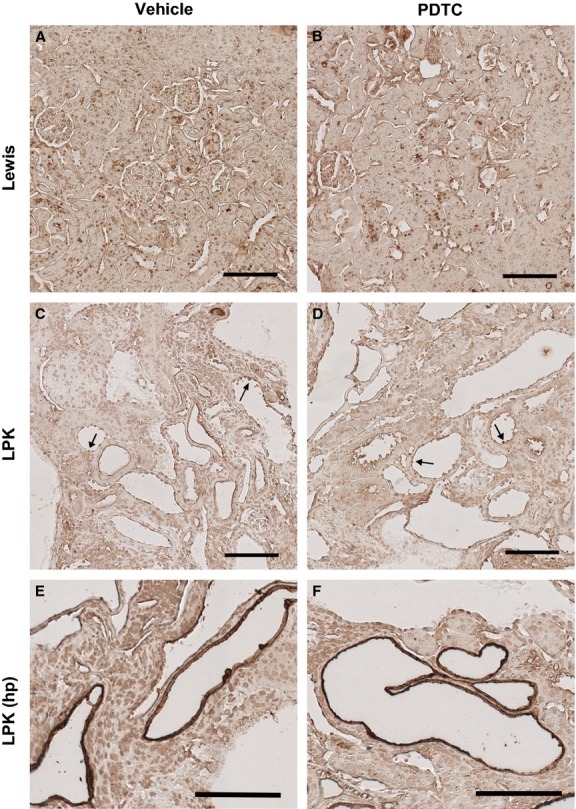
Effect of PDTC on cortical p50 immunolocalization. The renal cortex is shown from a Lewis rat treated with vehicle (A) and PDTC(40 × 2) (B), and Lewis polycystic kidney (LPK) rats treated with vehicle (C, E) and PDTC(40 × 2) (D, F). Lewis cortices displayed p50 positivity in distal tubule nuclei. In LPK cortices, moderately positive staining was observed in the cytoplasm and nuclei of cystic epithelial cells (CECs) (arrows), and in distal tubule nuclei. There was occasional strongly positive staining in the CECs of focal cysts (E and F). Scale bar = 100 *μ*m.

Western blotting and immunohistochemistry were also performed for p50 (the end‐product of p105 cleavage). Western blotting of whole kidney nuclear extracts demonstrated that p50 protein was significantly higher in vehicle‐treated LPK compared to Lewis animals (Fig. 7 and Table 3), but was not altered in PDTC‐treated animals. By immunohistochemistry, p50 was diffusely expressed throughout the medulla and cortex of Lewis kidneys (Fig. 8A). In LPK rats, p50 was also diffusely expressed (weak‐moderate in intensity) in all kidney regions, including cysts and CECs (Fig. 8C). In addition, selected cortical cysts (approximately 5% of all cysts) were strongly positive for p50, as shown in the high‐power view in Figure 8E. However, the localization and intensity of p50 immunoreactivity were similar between LPK + vehicle and LPK + PDTC (40 × 2) (Fig. 8C–F). By quantitative image analysis, when p50 positivity was expressed as a percentage of total tissue (not including cystic areas), no differences were found among the groups (Table 3). When p50 was expressed as a percentage of total area (including cystic areas), there was a trend toward higher p50 expression in the LPK + vehicle group compared to the Lewis + vehicle group (*P* = 0.057, Table 3), but this was also not altered in LPK rats treated with PDTC.

**Table 3.** Effects of PDTC on renal NF‐*κ*B expression and activity in Lewis and LPK rats.

Parameter	Lewis+V	Lewis + P(40 × 2)	LPK+V	LPK+P(40 × 2)
%p50 stain/tissue	58.7 ± 12.3	59.6 ± 9.3	50.5 ± 9.6	55.1 ± 3.7
%p50 stain/area	52.6 ± 13.7	54.5 ± 9.5	27.0 ± 7.6	27.6 ± 3.5
p50 protein (fold‐increase above Lewis+V)	1.00 ± 0.23	1.34 ± 0.18	2.30 ± 0.77^*^	2.23 ± 0.85
p65 transcription binding (A_450_)	1.00 ± 0.09	1.00 ± 0.10	0.56 ± 0.25^*^	0.73 ± 0.22

A_450_; absorbance value at 450 nm; LPK, Lewis polycystic kidney; P(40 × 1), PDTC 40 mg/kg once daily; P(40 × 2), PDTC 40 mg/kg twice daily; V, vehicle.

Expression of p50 protein was assessed by immunohistostaining and by Western blot. Protein:DNA binding activity was assessed by p65 transcription binding assay. Data expressed as mean ± SD.

*P < 0.05 versus Lewis + V. In all experiments: Lewis + V (*n* = 3) and Lewis + P (*n* = 4). For Western blotting and p65 binding experiments: LPK + V (*n* = 7–9), LPK + P(40 × 2) (*n* = 7–9). For p50 immunohistochemistry experiments: LPK + V (*n* = 4), LPK + P(40 × 2) (*n* = 4).

### Effects of PDTC on renal cortical nuclear p65‐DNA binding activity

To further examine NF‐*κ*B activation, p65 was also assessed in DNA binding assays of renal cortical nuclear extracts. Unexpectedly, p65‐DNA binding activity was reduced in LPK + vehicle compared to the Lewis + vehicle group (*P* = 0.04, Table 3). Moreover, there was a trend toward restoring p65‐DNA binding activity with PDTC treatment in LPK rats compared to LPK + vehicle (*P* = 0.09, Table 3).

### Effect of chronic bortezomib treatment on kidney enlargement and proteinuria in LPK rats

We furthermore assessed whether the proteasome inhibitor, BTZ, would have similar effects on kidney enlargement in LPK rats. Treatment with BTZ caused no adverse effects in LPK rats but did not alter the progression of kidney enlargement in LPK rats at week 6 (KW:BW of LPK + Vehicle: 3.27 ± 1.35; LPK + BTZ: 2.93 ± 1.31%, *P* = 0.28) or at week 10 (LPK + Vehicle: 6.21 ± 0.81; LPK + BTZ: 5.80 ± 0.38; *P* = 0.27). BTZ also did not alter proteinuria at week 10 (*P* = 0.25 vs. LPK + vehicle, Table 4).

**Table 4.** Effects of bortezomib on renal function and proteinuria in LPK rats.

Parameter	LPK+Vehicle (week 6) *N* = 3	LPK+BTZ (week 6) *N* = 3	LPK+Vehicle (week 10) *N* = 8	LPK+BTZ (week 10) *N* = 9
Final BW	146 ± 16	139 ± 23	192 ± 16	188 ± 12
HW:BW (%)	–	–	0.496 ± 0.04	0.504 ± 0.04
Urine Pr:Cr (mg/mmol)	11 ± 4	11 ± 4	138 ± 58	105 ± 58
24‐h urine volume (mL)	5 ± 2	5 ± 3	20 ± 5	20 ± 6
Serum urea (mmol/L)	6 ± 2	8 ± 3	12 ± 2	14 ± 3
Serum Cr (*μ*mol/L)	22 ± 2	26 ± 2^*^	35 ± 2	39 ± 5^†^
Serum Albumin (g/L)	26 ± 1	25 ± 1	28 ± 2	26 ± 2
CrCl/BW (*μ*L/min/g BW)	5.1 ± 0.7	4.4 ± 0.9	4.5 ± 0.6	4.1 ± 0.5

BW, body weight; BTZ, bortezomib; Cr, creatinine; CrCl/BW, creatinine clearance corrected for body weight; LPK, Lewis polycystic kidney; HW:BW, heart weight to body weight ratio; Pr:Cr, urinary protein to creatinine ratio.

Data expressed as mean ± SD.

*P < 0.05 versus Lewis + vehicle (week 6), ^†^*P* < 0.05 versus LPK + vehicle (week 10).

## Discussion

This study investigated the chronic effects of PDTC in a rodent model of PKD. The key findings were that PDTC: (1) attenuated the progression of TKV and cyst volume as determined in serial MRI scans; (2) reduced kidney enlargement, as assessed by ex vivo measurement of the KW:BW ratio; (3) decreased the progression of proteinuria; and (4) did not alter the decline in renal dysfunction, markers of chronic interstitial injury (including monocyte accumulation, renal cell proliferation, and interstitial fibrosis) or significantly alter the expression or activity of NF‐*κ*B proteins at the final time point of the study.

Previous experimental studies have shown that PDTC attenuates kidney enlargement in nephrotic glomerular diseases (Rangan et al. 1999; Tapia et al. 2008) and renal cancer (Morais et al. 2010). To our knowledge, the current study is the first to demonstrate that PDTC attenuates the progression of kidney enlargement secondary to PKD. In the current study, serial MRI assessment of the same animal confirmed that the TKV at the commencement of treatment was similar in both LPK rat groups. Region‐based threshold analysis of MR images (Lee and Lee 2006; Reichardt et al. 2009) showed that cyst volume was attenuated with PDTC treatment compared to the vehicle group in LPK rats, confirming that the decrease in kidney enlargement was mediated through the attenuation of cyst growth.

In contrast to the decrease in cyst volume shown in MRI, there was no change in the percentage cyst area in histological sections. In the LPK model, cysts are diffuse and account for the majority of the histological section area. One possible reason for the discrepancy between histologically measured area and MRI‐assessed TKV, is that both the absolute cystic and interstitial areas decreased proportionally in PDTC‐treated kidneys, and thus the overall percentage cyst area assessed in histological sections did not change. Furthermore, histological analysis occurs only in the 2‐dimensional plane and may also underestimate cyst size compared to MRI, due to fluid loss or tissue shrinkage during histological fixation (Wallace et al. 2008).

Cystic growth in PKD is due to a combination of increased cellular proliferation, abnormal planar cell polarity and dysregulated trans‐epithelial fluid transport (Happe et al. 2009). Renal cell proliferation was increased 4.6‐fold in vehicle‐treated LPK rats in this study and was not affected by PDTC treatment. Previous work has identified that renal cell proliferation peaks at week 3 in LPK rats (Phillips et al. 2007; Schwensen et al. 2011), therefore it is possible that PDTC altered renal cell proliferation at an earlier time point in the present study.

Pyrrolidine dithiocarbamate reduced proteinuria in LPK rats, as shown in other renal disease models (Sakurai et al. 1996; Tapia et al. 2008). Similar to these earlier studies, the anti‐proteinuric effect of PDTC in LPK rats was partial, being evident at week 10 and only reaching statistical significance in the 40 mg/kg twice daily group. The mechanisms underlying the anti‐proteinuric effect of PDTC in LPK rats are yet to be elucidated. In protein‐overload nephropathy, Mudge et al. suggested that PDTC decreases proteinuria by antioxidant‐mediated suppression of NF‐*κ*B activation within podocytes (Mudge et al. 2001). The persistence of tubulointerstitial disease in LPK rats implies that PDTC probably did not decrease proteinuria by restoring impaired tubular reabsorption. Heavy proteinuria is associated with hypertension and worse renal function in ADPKD (Chapman et al. 1994), but the implications of the anti‐proteinuric effects of PDTC in this disease are unclear.

Pyrrolidine dithiocarbamate did not improve renal dysfunction in LPK rats. This is probably because cystic microarchitecture remained severely abnormal in PDTC‐treated rats and also because the reduction in kidney enlargement by PDTC was only partial (~25%). The dose and route of PDTC administration were determined according to previous studies (Rangan et al. 1999). In preliminary studies, a single intraperitoneal dose of PDTC higher than 40 mg/kg caused neurotoxicity in LPK rats, similar to previous data (Rangan et al. 1999). To exclude the possibility that a higher total daily dose would not be more efficacious, we also administered PDTC 40 mg/kg twice daily, but this achieved minimal additional reduction in KW:BW ratio over the 40 mg/kg once daily group. Another caveat of this study is the initiation of PDTC administration at an early phase of disease. Whether PDTC decreases kidney enlargement if commenced at a later stage of disease requires further evaluation.

Because PDTC is a classical inhibitor of the NF‐*κ*B signaling pathway, this study assessed the expression and activation of two NF‐*κ*B proteins: p50 (through immunohistochemistry and Western blotting for p50, and immunohistochemistry for its precursor P‐p105); and p65 (by protein:DNA binding). By Western blot analysis, the expression of p50 in nuclear extracts was increased in the LPK model compared with Lewis. Consistent with this, and concurring with our previous studies (Ta et al. 2012), P‐p105 was highly expressed in the CECs of LPK rats. Additionally, p50 was localized to CECs and upregulated in focal cysts. However, quantitative histological analysis of p50 positivity as a proportion of tissue (%p50 stain/tissue) failed to reveal a difference in p50 positivity between LPK and Lewis kidneys. This suggests that p50 activation and expression are elevated in LPK compared to Lewis, although this difference is difficult to quantitate by immunohistochemistry since p50 is specifically localized to the CECs, which form only a small fraction of the histological tissue.

Our study found that p65:DNA binding was decreased in LPK kidneys compared to Lewis. This result was unexpected, since (1) the p65 subunit (typically heterodimerized with p50) is associated with upregulation of proinflammatory gene transcription (Hayden and Ghosh 2012); and (2) previous immunohistochemistry studies have demonstrated high expression of p65 in the CECs of *Pkd1−/−* mice (Qin et al. 2012). Overall, the data highlight differential activation of p65 and p50 in the LPK model, suggesting that while p50 activation is upregulated in LPK kidneys, there may be impairments in the nuclear transport or the DNA binding capacity of the p65 subunit (Neumann et al. 1995). Notably, NF‐*κ*B proteins can stimulate or repress transcription depending on the composition of the dimer formed (May and Ghosh 1997), thus further characterization of the NF‐*κ*B complexes in PKD is necessary.

NF‐*κ*B critically depends on the degradation of I*κ*B by the proteasome (Hayden and Ghosh 2012). Previously, proteasomal inhibition by carfilzomib reduced cystic disease in a murine model of PKD (Fedeles et al. 2011). However, our study found no improvements in KW:BW with BTZ, possibly suggesting that proteasomal inhibition alone is insufficient to reduce cystic disease in the LPK model. PDTC is thought to inhibit NF‐*κ*B by preventing I*κ*B degradation (Liu et al. 1999) or by preventing the dissociation of the cytoplasmic NF‐*κ*B: I*κ*B complex (Schreck et al. 1992). Contrary to our expectation, PDTC did not significantly decrease renal NF‐*κ*B expression or activity. It is possible that PDTC reduced p50 activation at an earlier time point in the study, since previous immunohistochemistry data (Ta et al. 2012) have indicated that P‐p105 expression is highly elevated at week 3 in LPK compared to Lewis kidneys. Of note, there was a trend toward higher p65‐DNA binding in PDTC‐treated LPK, however further studies are required to elucidate the significance of these findings. Alternatively, PDTC may have acted via non‐NF‐*κ*B signaling pathways, for example, through metal chelating or anti‐oxidative actions (Schreck et al. 1992).

Divergent to the current findings, Torres et al. reported that a dialkyldithiocarbamate derivative, sodium diethyldithiocarbamate (DDTC), aggravated disease in Han:SPRD rats, increasing kidney enlargement, cystic disease, plasma urea, and interstitial inflammation (Torres and Bengal 1995). The authors attributed these effects to the pro‐oxidant properties of DDTC in inhibiting metal superoxide dismutases. Compared to DDTC, PDTC has a higher stability at physiological pH (Cvek and Dvorak 2007) and therefore is less rapidly converted to the toxic degradation product, carbon disulfide (Chabicovsky et al. 2010). PDTC can induce neurological and hepatic toxicity at high doses and is currently not approved for human use, but is undergoing preclinical evaluation as an anti‐viral agent (Chabicovsky et al. 2010).

In conclusion, chronic PDTC administration reduced the progression of kidney enlargement, cyst volume, and proteinuria, but did not alter renal function, interstitial injury or NF‐*κ*B activation in a nongenetically orthologous model of PKD. Future studies should verify the efficacy of PDTC in genetically orthologous models of ADPKD (in combination with other agents and at different disease stages), and should also investigate the mechanism/s of action of PDTC in suppressing cyst growth in in vitro models.

## Acknowledgments

The authors thank V. James, K. Schwensen, K. Byth, E. Diefenbach, and D. Liuwantara for their assistance with histology, statistical analyses, NF‐*κ*B DNA binding assays, and Western blotting, respectively.

## Conflict of Interest

The authors declare that they have no competing interests.
